# Understanding Autonomous Shuttle Adoption Intention: Predictive Power of Pre-Trial Perceptions and Attitudes

**DOI:** 10.3390/s22239193

**Published:** 2022-11-26

**Authors:** Fahimeh Golbabaei, Tan Yigitcanlar, Alexander Paz, Jonathan Bunker

**Affiliations:** 1 School of Civil and Environmental Engineering, Queensland University of Technology, 2 George Street, Brisbane, QLD 4000, Australia; 2 City 4.0 Lab, School of Architecture and Built Environment, Queensland University of Technology, 2 George Street, Brisbane, QLD 4000, Australia

**Keywords:** autonomous vehicles, driverless car, shared demand-responsive transit, autonomous demand-responsive transport, autonomous shuttle bus, user acceptance, adoption intention, technology acceptance model, South East Queensland, Australia

## Abstract

The capability of ‘demand-responsive transport’, particularly in autonomous shared form, to better facilitate road-based mobility is considered a significant advantage because improved mobility leads to enhanced quality of life and wellbeing. A central point in implementing a demand-responsive transit system in a new area is adapting the operational concept to the respective structural and socioeconomic conditions. This requires an extensive analysis of the users’ needs. There is presently limited understanding of public perceptions and attitudes toward the adoption of autonomous demand-responsive transport. To address this gap, a theory-based conceptual framework is proposed to provide detailed empirical insights into the public’s adoption intention of ‘autonomous shuttle buses’ as a form of autonomous demand-responsive transport. South East Queensland, Australia, was selected as the testbed. In this case study, relationships between perceptions, attitudes, and usage intention were examined by employing a partial least squares structural equation modeling method. The results support the basic technology acceptance model casual relationships that correspond with previous studies. Although the direct effects of perceived relative advantages and perceived service quality on usage intention are not significant, they could still affect usage intention indirectly through the attitude factor. Conversely, perceived risks are shown to have no association with perceived usefulness but can negatively impact travelers’ attitudes and usage intention toward autonomous shuttle buses. The research findings provide implications to assist policymakers, transport planners, and engineers in their policy decisions and system plans as well as achieving higher public acknowledgment and wider uptake of autonomous demand-responsive transport technology solutions.

## 1. Introduction

The exponential technological advancements that we are currently experiencing, particularly in artificial intelligence (AI), are providing opportunities for disruption in many sectors [1]. The transport sector is one such sector. With advances in AI, there is a great promise that driverless cars or autonomous vehicles (AV) will address some of today’s transport challenges e.g., facilitating mobility for the transport-disadvantaged population, enhancing mobility efficacy, improving safety, reducing emissions [2,3], accelerating freight transport [4,5] and enabling underwater transport [6]. Emerging business models, like Shared-AVs (SAVs) which are capable of ridesharing in various ways, will make it easier to provide demand-responsive services on non-fixed routes [7,8]. Examining the likelihood of adoption and usage intention of AVs is critical, as their integration into the transportation system has the potential to affect passengers’ mobility behaviors and lifestyles [9,10]. However, as AVs are not yet commercially available in the transport system, with the exception of a few trials, forecasting the precise travel demand is challenging in this domain in terms of, for example, ownership trends, preferred mode choices, and vehicle kilometers traveled (VKT). There might also be reactions to the introduction of this technology in the public domain [11,12].

A public transport system’s success relies on its ability to attract and retain passengers. The autonomous shuttle bus (ASB) as a form of shared autonomous demand responsive transit (ADRT) serves multiple passenger trips at a time [13] and has the potential to complement other means of travel including regular public transport (RPT), thereby increasing public transport usage overall [14]. It is worthwhile exploring the feasibility of this technology in terms of potential societal advantages. An ASB could have the potential to operate as a feeder to RPT, particularly in peri-urban regions, where first/last-mile access to public transit is challenging [15].

ASBs could also offer personal transport to individuals who do not have a driver’s license due to aging, or medical ailments—i.e., physical and/or cognitive disabilities [16,17,18]—or children [19]. ASBs are currently at the prototype stage in several projects operating on predetermined routes at restricted speeds. They typically accommodate between 8 and 10 passengers and need to be supervised by either an onboard steward or an external control room. Many substantial challenges are yet to be overcome for the ASB concept regarding perceptions of the general public, policies, and traffic management. Individuals’ perceptions and adoption intentions toward ADRT are important as they influence demand for such technologies, governance policies, and future infrastructure investments [20].

Public perception has been defined as “the type of information obtained from a public opinion survey, which is merely the aggregate views of a group of people (usually a randomly selected sample) who are asked directly what they think about particular issues or events” [21]. It could be interpreted as “the difference between an absolute truth based on facts and a virtual truth shaped by popular opinion, media coverage and/or reputation” [22]. Responses to “structured questions can be recorded and analyzed in simple, quantitative terms as a sort of snapshot of opinion at a given moment in time” [21]. The requirements of different individuals can be identified via their perceptions because public perceptions are affected by their demands. It is essential to recognize our perception because it is the driving force behind our reaction to things [23].

While many surveys have recently been conducted on the public’s perception of AVs [23,24,25,26], limited studies thus far have specifically explored whether the public view ASBs as a viable alternative to their existing modes of travel [27,28,29,30,31,32,33,34,35,36,37]. Acheampong et al. [15] state that “research on technology and innovation acceptance and diffusion, and choice behavior under volitional control do provide various theoretical models”, which can be used as an argument for the investigation of the adoption of such kinds of demand-responsive services. By specifying the interrelationships of the psychological variables of theoretical models, we would be able to provide more systematic predictions about user adoption behavior and gain deeper insights.

Generally, behavioral theories include a variety of psychological factors that are considered potential predictors of behavioral intention. Additionally, the inclusion of behavioral theories allows researchers to progressively uncover the underlying correlations between psychological factors, ensuring that plausible and profound findings can be presented [38]. Ultimately, these comprehensive empirical insights would enlighten travel-demand modeling and management strategists.

**Figure 1:**
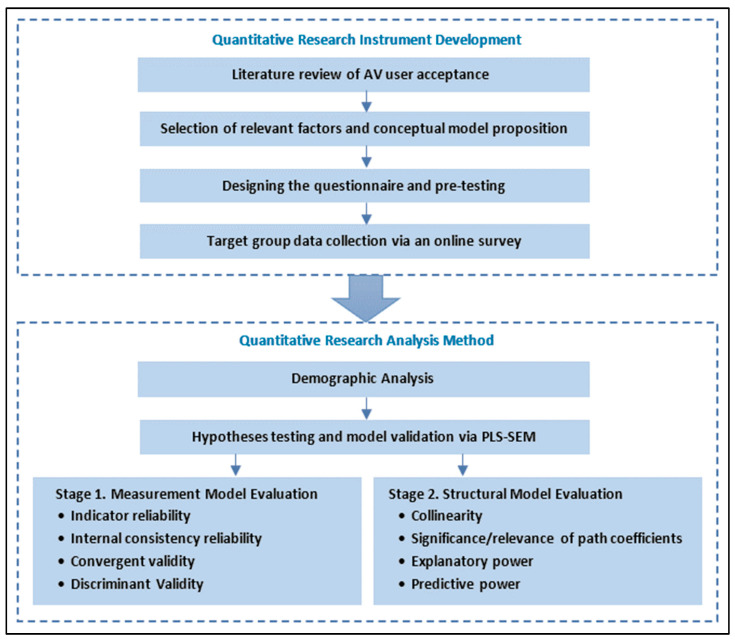
Study design flowchart.

This research contributes to developing a conceptual framework by using common hypotheses from the Technology Acceptance Model (TAM) [39] to explore the psychological factors and their interrelationships, which can affect adult travelers’ perceptions and adoption of ADRT options, particularly ASBs as a public transport mode in South East Queensland (SEQ), Australia. The comprehensive insights provided by this research can assist policymakers, transport planners, and engineers in their policy decisions and system plans as well as achieving higher public acknowledgment and potentially wider uptake of ADRT technology solutions where appropriate. The layout of the study design is illustrated in Figure 1.

This paper is structured as follows. Section 2 reviews the theoretical background. Section 3 explains the research methodology, including the survey design, hypothesis development, and demographics of the survey participants. Section 4 focuses on the results of the partial least structural equation modeling (PLS-SEM) analysis including (a) measurement model evaluation, (b) structural model evaluation, and (c) hypotheses testing. Section 5 concludes by discussing the main findings and practical implications. Finally, we put forward the research limitations by recommending some further research plans in Section 6.

## 2. Theoretical Background

The ability to predict AV adoption intention is a relatively new concept. Several surveys, however, have been conducted to predict the future of AVs [25,29,30,32,40,41,42]. To explore the span of studies on determinants of public acceptance of AVs, we conducted a systematic review [12] and classified the influential factors on willingness to use this technology. In line with our findings, the Technology Acceptance Model (TAM) and its variants are the most commonly utilized underlying theory. TAM was first introduced by Davis [43] who adapted the Theory of Reasoned Action (TRA) [44] to explore/explain the determinants of usage/non-usage of technological innovations. Behavioral intention is recognized as a reliable predictor of actual usage, particularly in the context of studies on current and/or emerging technologies that are not yet commercially available [45,46,47]. According to Davis & Venkatesh [48], the “expectations about a system captured using reliable and valid measures of key expectations, even before hands-on use of the system, are predictive of those that would have been obtained after a brief use of a test prototype, as well as after several weeks of the actual system use”.

Davis & Venkatesh [48] evaluated TAM’s capability in the prediction of technology acceptance. Throughout their pre-design and development phases, they discovered that TAM theory is helpful to predict the adoption of technologies. Despite the emergence of other theories in this context, i.e., the unified theory of acceptance and use of technology (UTAUT), TAM continues to be the most robust approach and powerful technique for forecasting technology adoption in various domains [49], as it can predict nearly 40% of the variance among individuals’ behavioral intention and their actual behavior [50]. Therefore, TAM comprising its basic components is considered to be a suitable foundation, while adding constructs to already extended theories may introduce interferences [30].

Rahman et al. [51] assessed the utility of TAM, TPB, and UTAUT by applying a sample of 430 surveys. The outcomes of Hotelling’s *t*-squared test [51] for non-independent correlations indicated that all models were capable of effectively predicting driver acceptance in terms of behavioral intention; however, the original TAM model was noted to be the most accurate. Their findings support the idea that attitude toward a behavior is shaped based on relevant beliefs [52]. Perceived ease of use, identified as the belief in the simplicity of behavior, has two basic mechanisms to impact attitude: self-efficacy and instrumentality [39]. If the behavior is easier to perform using the technology, it will create a sense of efficacy and personal control for the performer.

Additionally, an easier system would contribute to enhanced performance with the same amount of effort. The enhancement of performance corresponds with the belief of usefulness (perceived usefulness); though, with its self-efficacy mechanism, perceived ease of use influences attitude above and beyond perceived usefulness. However, in the UTAUT model, the mediation of attitude on how personal beliefs (perceived usefulness and perceived ease of use) affect behavioral intention was overlooked, and perceived usefulness and perceived ease of use were only reflected as predicting variables of Behavioral Intention with simple linear regression. Subjective norms showed a positive, though a very small effect, on behavioral intention [51].

As was proven by Rahman et al. [51], considering the interpretations of these factors and the scales employed in prior research, it is clear that ‘performance expectancy’ is very similar to perceived usefulness in TAM, and ‘effort expectancy’ is very similar to perceived ease of use in TAM, and ‘social influence’ is very similar to ‘subjective norms’ in TPB. The high correlation between these pairs of factors and their comparable effects on behavioral intention supports statistical evidence of their similarity. UTAUT was only able to explain 71% of the variance in behavioral intention, the lowest percentage among the evaluated models. Along with this empirical evidence, UTAUT includes a total of eight factors (four components and four moderator variables), which is the highest number of factors among all the models, making the use of this model comparatively challenging. Due to its under-performance, the similarities with TAM and TPB, and the complex nature of the model, UTAUT was not considered to be useful for modeling the acceptance of ASBs in this study.

Regarding the TPB model, the design of both TAM and TPB is very similar, and both proposed three factors, one of which was shared by both: attitude. Hence, researchers had to consider the practical significance of adopting one model over the other. TAM provides a mechanism for explaining the formation of attitude, which was found to be the strongest of the factors in both models, by proposing that perceived usefulness and perceived ease of use can predict attitude. Of the TAM factors, perceived ease of use has the potential to provide actionable information to the developers of vehicle technologies and is not considered in TPB. TPB provides information on normative beliefs, behavioral control beliefs, and their effects on behavioral intention. The practical implications of these factors are less obvious [51]. Considering all these facts, the use of the TAM model to study the acceptance of ASBs could provide more actionable information and explain more variance in behavioral intention compared to the other models.

Extended TAM models have also been proven useful for assessing the public acceptability of new transport technologies. As depicted in Table 1, trust [24,25,29,40,41,42,53,54,55,56], perceived risk (safety, financial, socio-psychological, performance, privacy) [29,30,40,56,57], personality factors [25], relative advantage [29], social influence or subjective norms [25,29,40,53,55], sensation seeking [25], locus or desirability of control [25,29], ecological awareness [29,30], personal or consumer innovativeness [29,40], compatibility [40,41], perceived enjoyment [29,30], price evaluation [29,30,40], and self-efficacy [53,57] are found to be influential factors on AV adoption intention along with the basic TAM constructs.

Whereas prior research acknowledged influential factors on AV usage intention, some interrelationships between TAM constructs remained unclear. For instance, TAM suggests that the perceived ease of use influences the perceived usefulness and usage intentions, although some prior studies failed to support such cause-effect relationships [30,41,54]. In a similar vein, the misconception of AV systems caused inconsistencies in the previous studies of perceived risks. Xu et al. [24], Herrenkind et al. [29], and Lee et al. [57] conveyed that perceived risks negatively impacted AV usage intention, while Nastjuk et al. [40] stated that it did not. Without more investigation of the perceived risks of AVs, specifically ASBs, only a limited understanding of their prospective usage could be drawn.

Similarly, very few studies investigated the impacts of perceived relative advantages on the perceived usefulness and usage intention simultaneously [29,30,40]. Prior research was not also able to reveal the perceived relative advantages of ASBs compared to conventional counterparts. Since prospective users of AVs are customers of currently available modes, the impacts of the perceived relative advantages should be studied comprehensively. Some debates have arisen about whether perceived relative advantages and perceived usefulness need to be considered the same constructs [57]. Kulviwat et al. [59] reviewed the prior research and concluded that the two notions are contextually distinct and that perceived relative advantages are a predictor of perceived usefulness. Correspondingly, prior research has failed to explore the influence of perceived service quality in terms of ADRT acceptance, specifically ASBs. Even though this factor has been a focus in public transport acceptance research [60,61], research may have underestimated its impact on users’ perception, attitude, and usage intention.

**Figure 2:**
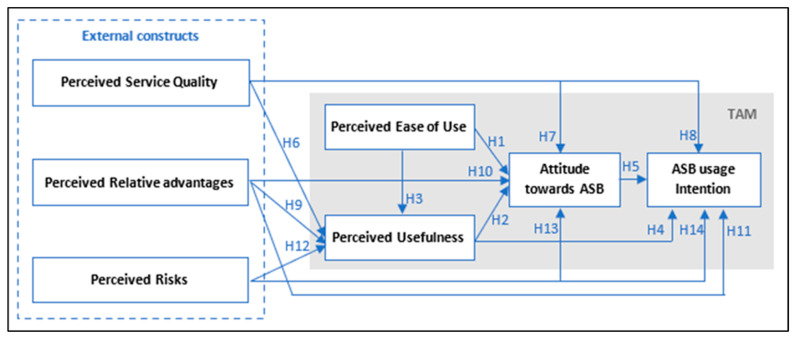
The conceptual research model for the ASB acceptance investigation.

Travelers are only starting to consider using ASBs if they perceive this new demand-responsive mode would offer enhanced service quality. To address this issue, and given that the belief and motivation towards using new technology may be influenced by more stimuli [43], this study proposes an effective conceptual research model for public acceptance of ASBs based on public passenger transport characteristics and focusing on constructs that are most associated with public transport usage, namely perceived service quality, perceived relative advantage, and perceived risks, in addition to the four basic TAM constructs for the sake of investigating the interrelationships between these key factors in the concept of ASBs, and increasing the context-specific clarity of prediction of adoption intention, and thereby actual usage behavior, accordingly gaining greater insight for policymaking, as “identifying the strongest concerns relating to ASBs can assist in the planning of proactive efforts to address these issues while building on any perceived positive attributes” [23,62]. The proposed conceptual model is illustrated in Figure 2.

## 3. Research Model Hypothesis Development

### 3.1. Technology Acceptance Model

As AVs are not yet commercially available in the market to use, and as our conceptual model is founded on TAM, we chose usage intention instead of actual use as the primary endogenous (dependent) factor to study end-users’ opinions of ASBs [63,64]. Usage intention can be interpreted as the extent to which an individual is willing/ready to utilize an innovation [39,65]. In earlier research about AV acceptance, the positive impact of perceived usefulness on individuals’ usage intention was confirmed [25,41,66]. In contrast, literature about AV acceptance has conveyed an unclear connection between perceived ease of use and usage intention [67]. Many studies reported a positive influence of perceived ease of use on usage intention [24,25], though others could not identify a substantial effect [27,41].

Following the above-mentioned findings, we assume that individuals who consider ASBs to be useful and effortless to use would have more positive attitudes towards them, and, consequently, will be more expected to intend to prioritize using ASBs over cars when this mode becomes available (i.e., usage intention). Subsequently, we postulate the following hypotheses:

**Hypothesis** **1** **(H1):** Perceived ease of use positively impacts attitude towards use of ASBs.

**Hypothesis** **2** **(H2):** Perceived usefulness positively impacts attitude towards use of ASBs.

**Hypothesis** **3** **(H3):** Perceived ease of use of ASBs directly impacts its perceived usefulness.

**Hypothesis** **4** **(H4):** Perceived usefulness of ASBs positively impacts the users’ usage intention.

**Hypothesis** **5** **(H5):** Attitudes towards ASBs positively impact the users’ usage intention.

### 3.2. Perceived Service Quality

In the marketing/management context, service quality is considered to be a key component in investigating customers’ behavior toward a product/service [68]. Perceived service quality is “the subjective assessment of customers about the overall perfection or superiority of a product/service” [69,70]. Customers’ evaluations of services vary according to their expectations or perceptions of service quality [71,72] concerning technical/functional service aspects [73]. Frequently, perceived quality does not correspond to the real quality of the product/service. The perceived quality is influenced by the customer’s assessment, while the actual quality is defined by product/service orientation [74]. If customers perceive a higher level of service quality, their intention to use/purchase a product/service is likely to rise [70].

In the transport domain, service quality is related to particular service characteristics, such as frequency, cleanliness, comfort, speed, accessibility, timeliness, information, and safety [75,76]. Public transport system service quality has been extensively investigated as a predictor of customer satisfaction or loyalty [77]. Recently, research has observed the link between perceived service quality and usage intention in the public transport context. Enoch et al. [60] and de Ona [61] studied the prospective involvement of DRT systems in sustainable mobility. They indicated that the primary factors determining user adoption of DRT are the range of destination coverage, ease of access to service, availability of comprehensive reliable information, ease of booking, service on-time performance, and affordable fare rates [78].

Perceived quality should be a high-priority concern for both transport policymakers and DRT service providers. Despite the fact that perceived service quality has been the focus of several public transport studies, its impact on the formation of passengers’ attitudes towards ADRT services and consequently intention to use ASBs have not received much attention. Therefore, we hypothesize:

**Hypothesis** **6** **(H6):** Perceived service quality of ASBs positively impacts their perceived usefulness.

**Hypothesis** **7** **(H7):** Perceived service quality of ASBs positively impacts attitude towards them.

**Hypothesis** **8** **(H8):** Perceived service quality of ASBs positively impacts intention to use them.

### 3.3. Perceived Relative Advantages

The perceived relative advantage of such mobility services is another aspect of ADRT research that is worth considering. The perceived relative advantages notion is “a characteristic of innovation, described as how an innovation is evaluated in comparison to its previous manifestation or idea” [30]. Kulviwat et al. [59] identified the link between perceived relative advantages and perceived usefulness while describing their distinction in the consumer context. Individuals’ perception of the usefulness of innovation would be higher when they perceive it as being superior to its precursor. Perceived relative advantage is positively associated with attitude towards use behavior, as consumers are comparing and contrasting an innovation’s attributes, which possibly influence their adoption decision [29,40].

In this context, perceived relative advantage is interpreted as how much ASBs are perceived to be superior to conventional shuttle buses [40,79]. This depends on whether the public views ASBs as being advantageous. It has been proven that the perceived relative advantages positively affect public preferences toward AVs [80]. Schoettle & Sivak [81] discovered that 57% of survey participants had positive attitudes towards AVs, primarily owing to their perceived relative advantage. Talebian & Mishra [5] also stated that the perceived relative advantages of AVs influence their adoption and diffusion. Following the above-mentioned findings, we hypothesize:

**Hypothesis** **9** **(H9):** Perceived relative advantage of ASBs positively impacts their perceived usefulness.

**Hypothesis** **10** **(H10):** Perceived relative advantage of ASBs positively impacts attitude towards them.

**Hypothesis** **11** **(H11):** Perceived relative advantage of ASBs positively impacts intention to use them.

### 3.4. Perceived Risks

Within technological innovation research, perceived risks are a major construct in the prediction of intention toward adoption [9]. Perceived risk can be defined as “a complex feeling of worry, fear, and anxiety that originates from a nervous situation” [70,82]. Individuals accept or reject a technology based on their beliefs and expectations, that is, perceived usefulness (benefits) and perceived risks. This concept is termed a risk–benefit paradigm in behavioral decision research and is deemed to be the extended bounded rationality notion.

After reviewing the existing literature, the following were found to be the top reasons among potential users being unwilling to use AVs of any type [12]; perceived risks regarding safety issues, equipment/system breakdown, performance in mixed mode traffic situations and interactions with other road users, cyber security and hacking threats, data privacy, and lack of control during accidents. Most of the respondents preferred the vehicle to be supervised by a ‘human override’ as they thought that human performance is better in the case of instant decision-making.

However, some researchers, e.g., Liu et al. [83,84], reported an insignificant impact of perceived risks on usage intention caused by either respondents’ preferential risk tolerance for perceived advantages, or their lack of understanding of AV risks, while Xu et al. [24] confirmed its negative effects. Acheampong & Cugurullo [85] reported a positive correlation between perceived risks (fears and anxiety) regarding AV systems and the perceived usefulness of AVs and advocated that peoples’ concern about AV systems’ performance or their interactions with other road users does not essentially weaken their perceived usefulness. The reported contradictions in the findings call for more investigation into the role of perceived risks in the adoption of ASBs. So, we hypothesize:

**Hypothesis** **12** **(H12):** Perceived risks and perceived usefulness of ASBs are positively related.

**Hypothesis** **13** **(H13):** Perceived risks about ASBs negatively impact attitudes towards them.

**Hypothesis** **14** **(H14):** Perceived risks about ASBs negatively impact their usage intention.

## 4. Research Methodology

### 4.1. Survey Design

We followed the measurement development steps outlined in prior research [86,87]. The measurement indicators of each construct of the conceptual model (perceived relative advantage, perceived service quality, perceived risks, perceived ease of use, perceived usefulness, attitudes, and usage intention) were adapted and confirmed over a three-phase process. Initially, the research in transport/social science literature related to AVs was systematically reviewed to determine the measurement indicators for each research model component. The related indicators were measured by applying a five-level Likert scale. For a clearer interpretation of each shuttle type’s typical usage scenario, an introductory paragraph including some photographs of the shuttles was depicted at the beginning of each section of the questionnaire.

In the second step, the list of the measurement items and the introductory paragraphs were refined through consultation with an expert supervisory review panel representing the views of key informants in the field, specifically civil-engineering and built-environment academics specializing in transport systems and autonomous vehicles. An overview of theoretical constructs, their measurement indicators, and adapted references are listed in Table 2.

The remainder of the survey collected respondents’ socio-demographic characteristics. The survey’s written language was English. In the third step, a pilot study (pre-test) was performed to detect any potential errors or misunderstandings [86]. A 20-50 respondent sample size was deemed satisfactory with which to obtain feedback to help in identifying possible inconsistencies [90]. We targeted higher degree research students (HDR) and the staff of Queensland University of Technology (QUT) because these people usually have broader knowledge regarding the application of surveys for reliable results.

Accordingly, the survey link was directly sent to the Faculty of Engineering HDR and staff email addresses, which were targeted with the assistance of the university administration. Consequently, a convenience sample of 40 completed answers was collected. The pilot survey enabled us to assess the clarity of question items, to make sure that they were understandable in light of the study objective, and to achieve a satisfactory number of indicator items according to respondents’ input. We evaluated the total scale/sub-scale consistency and reliability of the question items by calculating Coefficient Alpha using the pilot survey results. We removed the items that degraded the reliability by analyzing the relative contribution of each item to overall scale reliability. After applying this approach, the survey questionnaire was finalized.

### 4.2. Case Study Region

A case study approach was adopted to explore the public adoption of shared DRT services, specifically conventional/autonomous shuttles, by adult residents of the SEQ metropolitan region, which is centered on Brisbane (Australia). SEQ has an adult population of approximately 3,650,000 people. The region was selected mostly because it is a highly car-dependent region, which raises transport disadvantage concerns. The region, therefore, “produces unique travel behaviors as a result of its mono-centric physical structure that created a high dependency between suburban areas and the central business district (CBD)” for trades, services, and facilities [91].

SEQ has 12 contiguous local government areas (LGAs), where each is a municipality administered by the third, and lowest, tier of government. The survey respondent recruitment methodology involved only individuals living within the urban and peri-urban areas of SEQ. Rural areas were excluded from the study because we expect that offering this service would not be cost-efficient due to its very low-density development and likely low patronage.

### 4.3. Data Collection Procedure

The finalized questionnaire, which was approved by the University Human Research Ethics Committee (UHREC RN: 2000000747), was hosted online and could be self-completed. To respond to the restrictions and risks due to the COVID-19 pandemic, Qualtrics (a professional web-based survey platform provider) was hired to reach out to the target respondents to compile data through a convenient random sampling method for this study. The survey link was passed to the general public by sending an e-mail to each potential respondent. The e-mail explicitly stated a brief description of the project and participant requirements, being adult residents of South East Queensland (SEQ). Data were collected during May 2021, with a total of 357 completed questionnaires. According to Krejcie & Morgan [92], the sample size for a population above 1,000,000 (confidence = 95% and Margin of Error = 6%) is 300. Tabachnick and Fidell [93] also advise that “it is comforting to have at least 300 cases for factor analysis, however, a smaller sample size (e.g., 150 cases) should be sufficient if solutions have several high loading marker variables (above 0.8)”. Stevens [94] denotes that “the sample size requirements advocated by researchers have been reducing over the years as more research has been done on the topic”.

### 4.4. Survey Participants

Ultimately, 300 valid responses with no missing value, invalid observation, or outliers were deemed to be satisfactory for further analysis after screening and cleaning the data. Table 3 illustrates the descriptive summary for each demographic group and an assessment of their multicollinearity. Results show that variable inflation factors (VIFs) are all acceptable, at a level of <2.50 [95].

Of the 300 survey participants, the 18–35 years-old age group was the highest proportion (36%) while 17.7% of the respondents were aged 36–50 years old, the remaining 19.3% and 27% accounted for respondents aged 51–65 and over 66 years-old, respectively. Female respondents were nearly double that of males (65% compared to 35%). Regarding education, 27.4% of participants held tertiary degrees and, of the remainder, 36.3% completed high school, while 36.3% held a vocational certificate. The retired, homemaker or not employed group accounted for 46% of respondents while part-time or casual employees accounted for 24% and full-time or self-employed 30% of the remainder. The median and mode annual income bracket was $52,000–$77,999. The survey also showed that two-thirds of respondents (67%) were living in peri-urban areas, and most of these were from two-person households (39.7%).

## 5. Data Analysis and Results

This research proposed 14 hypotheses exploring causal relationships amongst seven constructs: perceived service quality, perceived relative advantages, perceived risks, perceived ease of use, perceived usefulness, attitudes towards ASB, and usage intention.

According to Hair et al. [96], structural equation modeling (SEM) is the best analysis method to “map paths to many dependent (theoretical or observed) variables in the same research model and analyses all the paths simultaneously rather than one at a time” [97]. Covariance-based SEM (CB-SEM) analytically focuses on shared variance and confirms theoretically assumed relationships, while the partial least squares (PLS-SEM) method is applied for prediction and/or identification of relationships between constructs [70]. PLS-SEM is a variance-based technique that employs total variance in the estimation of parameters [98]. We considered PLS-SEM to be preferable to CB-SEM for the current study in the following respects, which concur with research in the same context [29,30,40,47,70,99].

PLS-SEM is statistically more powerful than CB-SEM and is appropriate for data with non-normal or unknown distributions as it is distribution-free [100]. This indicates that PLS-SEM can identify causal connections in the populations [101], making it easier to perform exploratory analysis for theory development [98,102,103] in the case of present work (an extension of an existing structural theory of TAM). Since the proposed conceptual framework is prediction-oriented, which seeks to offer a causal overview of the relationship between pre-trial perceptions/expectations and adoption intention, PLS-SEM is deemed to be the proper approach for studying complex latent interaction effects [100].

The two-step data analysis procedure recommended by [102] using SmartPLS 3.3.3 software [104] involved:

Measurement (outer) model evaluation: to check the structural reliability and validity of reflective (highly correlated and interchangeable) indicators and to modify the measurement model to develop a structural model to explain the relationship among the latent (unobserved) variables [105,106,107]; see Table 4.

Structural (inner) model evaluation: to evaluate the theoretical model and its associated hypotheses and to test whether the hypothesized interrelations among constructs within the measurement model are supported via the survey data [97], see Table 8.

According to Gefen et al. [108] applying measurement model and structural model assessment allow a combination of factor analysis and hypotheses testing to be combined in one operation.

### 5.1. Measurement Model Evaluation

In the PLS-SEM approach, measurement model evaluation is conducted by assessing three criteria, internal consistency reliability, convergent validity, and discriminant validity [98,109,110,111]. For the model calculation “using the PLS algorithm, the path weighting scheme is selected, with a maximum iteration number of 1000 and a stop criterion of 10-7” [108].

First, as illustrated in Table 4, we assessed internal consistency through composite reliability (CR) which should be over 0.7 [112], and Coefficient Alpha with a value > 0.7 [113]. The CR and CA values of all seven constructs are shown in Table 5 and range from 0.902 to 0.980, and 0.836 to 0.960, respectively, which are higher than the recommended thresholds, indicating decent internal consistency reliability. 

Second, we evaluated the convergent validity by checking the outer loading to be above 0.6 [96], the variance inflation factor (VIF) to be less than the adopted threshold of 5 [98], and the average variance extracted (AVE) is expected to be over 0.5, as recommended by Fornell & Larcker [109] and Bagozzi & Yi [114]. Table 6 shows loadings of all indicators that exceed 0.6. No item was removed owing to high VIF (see Table 6), indicating that there is no collinearity problem. Regarding AVE, all six constructs had AVE values between 0.555 and 0.797 (see Table 5), reaching the recommended standard of adequate convergent validity, indicating that all these constructs explain more than half of their indicators’ variance [98].

Third, discriminant validity, which is identified as “the extent to which a construct is truly distinct from other constructs by empirical standards” [111], is assessed by three criteria: the Fornell–Larcker criterion, Cross-loadings, and the heterotrait–monotrait (HTMT) criterion. The Fornell–Larker criterion refers to the “latent construct shares more variance with its assigned indicators than with another latent variable in the structural model” [109]. In statistical terms, the AVE of each latent construct should be higher than the construct’s highest squared correlation with any other latent construct [98].

Table 5 illustrates that the AVE value of each construct (ATT, PR, INT, PEU, PRA, PSQ, and PU) is greater than the respective construct’s correlation with other constructs in all seven cases. Concerning the cross-loading, “an indicator’s loading with its associated latent construct has to be higher than its loadings with all the remaining constructs” [95]. As depicted in Table 6, the outer loadings of all seven constructs are greater than their cross-loadings, and each item has a strong factor loading on its associated construct (*p* < 0.01). Henseler et al., [115] proposed the heterotrait–monotrait (HTMT) criterion as a value < 0.9 in order to test discriminant validity, which shows whether these are the same or different latent factors [100]. In Table 7, the HTMT values demonstrate a high level of discriminant validity.

In summary, the measurement model evaluation confirmed a satisfactory degree of internal reliability and validity of all seven constructs (ATT, PR, INT, PEU, PRA, PSQ, and PU) before performing the next stage of the structural model valuation. The standardized root mean square residual (SRMR) was 0.063, representing a good fit with respect to the cut-off value of 0.08 [115].

### 5.2. Structural Model Evaluation

First, the bootstrapping re-sampling procedure, as suggested by Chin [116] and Hair et al. [98] with *n* = 300 cases and *n* = 5000 resamples, was carried out corresponding to Hayes [117] to evaluate the structural model fit. The evaluation procedure involves examining the predictive power of the conceptual framework and analyzing interactions among the constructs.

**Figure 3:**
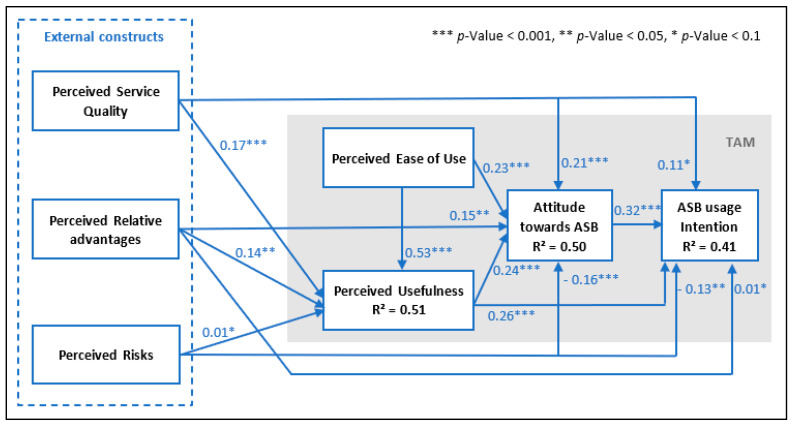
Structural model.

Next, the predictive power of the conceptual framework was assessed by checking two criteria: (a) The model’s predictive accuracy, evaluated by the coefficient of determination (R^2^) that indicates “the share of the variance of the exogenous variables affecting the endogenous variables. Range of R^2^ value is 0–1, but it needs to be at least 0.3 to be considered acceptable” [47] also see Table 8; (b) The model’s predictive relevance is evaluated by the Stone–Geisser Q^2^ [118,119], which signifies “an evaluation criterion for the cross-validated predictive relevance of the PLS path model” [102]. The outcomes demonstrated that PU, ATT, and INT were explained by the other constructs, with R^2^ values of 0.51, 0.50, and 0.41, respectively (see Figure 3).

According to the rule of thumb advised by Henseler et al. [103] see Table 8, the R^2^ value of perceived usefulness (PU) showed a greater level of predictive accuracy than the R^2^ value of attitude (ATT) and usage intention (INT), which represented a moderate level power to explain the generated variation amount [116]. Along with measuring R^2^, the Stone–Geisser Q^2^ value was estimated by performing the blindfolding technique—“a sample reuse technique that omits singular elements of the data matrix and uses the model estimates to predict the omitted part” [98]. As indicated by Hair et al. [111], “a measure of predictive relevance values of 0.02, 0.15, and 0.35 indicated small, medium and large predictive relevance for a certain endogenous construct”. The results showed that the Q^2^ of perceived usefulness (PU), attitude (ATT), and usage intention (INT) were 0.355, 0.378, and 0.305 respectively, which are significantly greater than zero, so had large predictive relevance. Thus, the predictive power of the current research conceptual framework was achieved for both R^2^ and Q^2^ values.

### 5.3. Hypothesis Testing

Next, the path relationship between the conceptual framework constructs was assessed by the standardized beta coefficient of ordinary least squares regression (β) that takes the value ≤|±1| and its significance. A β value near |±1|describes a strong effect on the latent variable, while a value close to zero describes a negligible effect. Values above 0.1 reflect significant influence [116]. The recommended values of large, medium, and small loading sizes are 0.5, 0.2, and 0.1 respectively [120]. The path coefficients were deemed to be significant when t-values were larger than |±1.96|at significant levels of 5% and confidence levels of 90% [111].

Accordingly, Table 9 reveals that all paths were significant except for three (Hypotheses H8, H11, and H12), indicating that PSQ and PRA have no direct influence on usage intention. Similarly, PR was shown to have no meaningful direct influence on PU. The outcomes also point out that PSQ has a stronger effect on PU than PRA, with β = 0.17 compared to 0.14. However, its effect on PU is still weaker than the influence of PEU on PU (β = 0.53). Out of five predictors of ATT comprising PU, PR, PRA, PSQ, and PEU, the results showed that PU has the strongest impact on ATT with β = 0.24. This was followed by PEU, PRA, and PSQ with β = 0.23, β = 0.21, and β = 0.15 respectively, while PR was the only predictor which influenced ATT negatively, with β = −0.16. Next, regarding the predictors of INT, ATT (β = 0.32) and PU (β = 0.26) were found to have a positive effect on it, whereas PR had a significant negative effect (β = −0.13).

## 6. Discussion of Findings and Implications for Policy and Practice

The following sections correspond to prospective policy implications that can be considered based on the hypothesis findings, which demonstrate how this methodology was used to identify practical implications.

### 6.1. Technology Acceptance Model

The study findings support TAM’s fundamental structure and demonstrate how ATT might remain to be a statistically substantial predictor of INT in the ASB context (H5). This is congruent with prior research in the domain, representing that a generally favorable attitude results in a higher usage intention and consequently actual use of ASBs [29] and AV [40]. The notable influence of PU on ATT (H2), which was confirmed by Herrenkind et al. [29] and Nastjuk et al. [40], and on INT (H4), which corresponds with [24,41] implies that the potential users might recognize ASB as a useful imminent travel mode.

The positive correlation of PEU with PU (H3), which was also verified by, e.g., Zhang et al. [25,56], and PEU with ATT (H1), in line with, e.g., Nastjuk et al. [40], indicate that, when individuals can utilize ASBs without experiencing significant physical or mental exertion, their impression of usefulness could be enhanced. These results are directly connected to prior research findings regarding users’ pre-trial beliefs about AVs (e.g., 40,42).

In contrast, some prior research has shown inconsistencies in the postulated relationships between the antecedents of TAM. For instance, the direct causality between PU and ATT could not be supported by Zhang et al. [56]. Similarly, PU failed to predict INT in the findings of Buckley et al. [54] and Herrenkind et al. [29]. In a similar vein, the association between PEU and PU was not confirmed. Henceforth, authorities should emphasize the efficacy and simplicity of ASBs in the technical improvement of impending mobility services. Policies could also definitely stimulate the public attitude. For instance, marketing campaigns can highlight the benefits of ASBs, portraying the mode as having more flexible timetables or consuming less energy than traditional buses [30].

### 6.2. Perceived Relative Advantage

Furthermore, the results showed a positive effect of PRA on both PU (H9) and ATT (H10), which has been shown in other research in both the ASB [29,30] and AV [40,57] contexts. This suggests that people who realize the advantages of ASBs over former conventional shuttle buses may find ASBs more useful and accordingly show greater adoption. However, understanding the link between perceived relative advantages and adoption attitude in the ASB setting is not enough and requires further work. Travelers may decide against using ASBs if they do not have a clear and distinct awareness of the benefits that only ASBs could bring. This is endorsed by the findings of Herrenkind et al. [29] regarding the insignificant association of PRA with ATT, and Lee et al. [57], who reported an insignificant association of PRA with INT, showing how individuals choose to adopt and utilize ASBs. They asserted that “the perception of usefulness at the psychological level was not influential on the intention to use but became influential at the system level” [57].

Consequently, highlighting the relative advantages could be a useful strategy to encourage travelers to make regular use of ASBs. To increase ASB usage intention, policymakers could accentuate not only the relative advantages over their conventional counterparts but also their inimitable usefulness. ASBs will be ecologically more sustainable than conventional shuttles because automated driving will improve the efficiency, safety, and flexibility of electrically powered ASBs, while also reducing driver errors, traffic congestion, and pollutant emissions [12]. As ASBs have a larger capacity than private cars, their widespread usage may help balance the growing mobility demand.

### 6.3. Perceived Risks

In alignment with Herrenkind et al. [29,30] in the context of ASBs, the results revealed the negative impact of PR on ATT (H13) and INT (H14), implying that perceived risk, as a psychological factor closely related to safety concerns, substantially lowering the potential users’ trust and usage intention. However, prior research on the impact of PR has shown contradictory findings in the AV context. For instance, while e.g., Xu et al. [24] failed to identify its significant effect on the INT, Zhang et al. [56], and Nastjuk et al. [40] reported its notable negative influence. Correspondingly, PR is predicted to negatively affect attitudes toward AVs [56] and usage intention indirectly by impacting users’ level of trust toward AVs. These inconsistent findings denote respondents’ difficulties in evaluating and estimating the implication of implementation of AVs due to the lack of practical evidence in various settings such as several road types, driving situations, or physical/mental conditions [57,121]. Therefore, the degree to which people perceive that ASBs have low risks determines whether or not they want to use this mode.

In addition, we could not find any association between PR and PU (H12). This may imply that, although the technical level of AVs is constantly improving, the market requirement for the technical stability and reliability of ASBs is also continuously increasing [89]. Given that concerns might unfavorably impact potential users’ decision-making, “policymakers must work to ensure that the general public understands the technology behind” ASBs [30]. The availability of comprehensive accurate information regarding the system’s decision-making procedure could be helpful in this regard. Offering trial rides may also assist build or reinforcing trust in ASBs since the initial experience of an ASB ride is probably associated with a feeling of fear/anxiety because control is transferred from the driver to the vehicle [30]. Nevertheless, such measures could lead to enhanced perceived trust in ASBs and reduced amount of risk perceptions, hence lessening undesirable impacts on usage intention.

### 6.4. Perceived Service Quality

Turning to the newly added construct, perceived service quality, the examination of the influence of this construct in association with perceptions regarding relative advantages, and risks, along with TAM constructs in the ASB domain has not yet been performed. Thus, our findings are unique and significantly contribute to the ADRT literature. The study revealed that PSQ has a positive direct influence on PU (H6) and ATT (H7). This finding is in alignment with previous public transport literature, e.g., Machado-Leon et al. [122] indicated that perceived service quality is an antecedent of perceived benefits, and it directly affects involvement. Accordingly, once potential users perceive they can travel in ASBs irrespective of time constraints and that the mode is compatible with their lifestyle or identity, they would find it more beneficial and consequently would show a more positive attitude toward their use.

Nevertheless, INT is not determined by PSQ (H8) according to the statistical results, even though PSQ is found to predict ATT (H7). As such, PU and ATT fully mediated the causal link from PSQ to INT, implying that perceived service quality indirectly affects ASB usage intention. This indirect association between perceived service quality and usage intention is consistent with other research outcomes in the public transport domain [123,124], suggesting that while users’ attitudes toward ASBs at the beginning of the employment stage will probably be positive, their usage intention may only increase with time and more awareness. However, these relationships are explored in the ASB context for the first time, contributing to extending our understanding of the importance of service quality perceptions in forming both attitudes toward ASBs and intention to use this mode regularly. Further investigation is yet needed to verify these hypotheses.

In order to improve the perceived service quality, authorities could potentially adjust the condition of the ASBs regarding comfort, convenience, and safety, as well as the time frame of the availability of services to the requirements of the residents, before deploying them into public transport service. The government might also consider marketing and/or the subsidization of fares to be affordable to complement the existing RPT modes.

## 7. Conclusions and Future Research

A transition to more sustainable transportation is critical, due to recent ecological trends. The rapid and widespread distribution of shared autonomous demand-responsive transit (ADRT) may directly contribute to this goal since autonomous vehicle (AV) technology promises to deliver ecological benefits as it evolves. Due to the potential for ADRT services, especially autonomous shuttle buses (ASBs), to complement public transport demand, both researchers and policymakers need to understand how prospective users’ behavioral intentions to utilize such services are formed in order to attract and keep consumers in the future. While the interrelationships among perceived relative advantages and perceived risks have been investigated in the autonomous public transit adoption literature, they have attracted less attention in the ASB context. In particular, the potential impacts of perceived service quality as a commonly used notion in both public transport and behavior research have been overlooked in the ASB domain, indicating a need for more exploration of a robust acceptance concept.

The commercial entities developing ASBs and government agencies responsible for ensuring appropriate physical and social infrastructures are in place are likely to be interested in the extent to which the general public is aware and supportive of the impending roll-out of ASBs. In particular, identifying the strongest concerns relating to ASBs can assist in the planning of proactive efforts to address these issues, while building on any perceived positive attributes [23,59,125,126]. Consequently, we developed a conceptual model and included the abovementioned factors as extra stimulating technology acceptance factors, mainly owing to the nature of the ASB services’ potential to be integrated with public transport, to find their interrelationships and weight in ASB adoption [127]. 

The findings reported in this study make a noteworthy contribution to the ADRT acceptance literature by demonstrating the usefulness of the method used to underpin public attitude and intention to use ADRT technology. The research findings exemplify managerial implications to assist policymakers, transport planners, and engineers in their policy decisions and system plans as well as achieving higher public acknowledgment and wider uptake of ADRT technology solutions. We also advocate the importance of stimulating technological innovation efforts through government incentives for further advancement of ASB technology to make them smarter and safer [128]. Notwithstanding the comprehensive empirical information presented in the current work, there are certain research limitations worth underlining for future study.

The authors note the potential for bias in the findings due to issues around the representativeness of the small-scale sample size to the SEQ regional population. Future research could adopt the methodology but conduct a more systematic approach befitting a large, cross-sectionally representative sample to lower the possibility of bias, facilitating the development of more robust policy implications.

The data collection was performed by conducting an online survey with potential selection bias. For instance, respondents with higher educational levels are better at using and responding to emails compared to tech-disadvantaged groups. Given that ASBs are not yet available in the market, an online survey is an effective approach to exploring public adoption intention. Nevertheless, further large-scale surveys are required to more broadly understand the public attitude. Particularly, there are other social-science theories, such as TPB, for conducting future research on this concept from a different point of view. Some longitudinal surveys could be used to track changing views and behaviors over time.

We have focused on safety risks but the effects of other aspects involving psychological, social, and privacy risks should be investigated in this context as well. Furthermore, trust toward ASBs and the perceived risks of using the mode should be examined jointly. This strategy could provide an in-depth insight into how to improve travelers’ adoption intention. Additionally, investigating attitudes toward trust and perceived risks without physically exposing survey participants to riding in ASBs in real traffic systems may lead to biased results as their perceptions may be shaped by information attained from mass media, particular literature, or entertainment venues. Hence, combining mainstream questionnaires with novel methodological techniques like virtual reality, simulation, or gamification [121] may be useful.

As ASBs facilitate mobility for transport-disadvantaged populations, such as the elderly, those with no driving license, and physically impaired persons, and helps in minimizing transport related social exclusion [129], these groups must be involved in future studies. Moreover, further exploration of the effects of demographic characteristics (e.g., age, gender, education, income, or residential location) that might associate with the adoption of ASBs is desirable. This would result in a more nuanced knowledge of a particular target group (subject to their features) and improve the predictive power of the suggested framework. Lastly, while the current study follows a quantitative method, we recommend using qualitative research approaches, such as interviews to further investigate and validate these study findings.

## Figures and Tables

**Table 1:** Prior studies on AV adoption based on TAM and its extensions.

Author	Focus	Data Collection Method	Analysis Method	Investigated Constructs	R^2^ (Variance Explained)
Zhang et al. [25]	AV	Online survey	PLS-SEM	TR, SI, Sensation seeking, Big Five personality, PU, PEU → INT	0.54
Nastjuk et al. [40]	AV	Qualitative research, online survey	PLS-SEM	SN, LOC, PPR, TR, EA, PI, RA, Co, enjoyment, PrE, PU, PEU, ATT → INT	n/a
Motamedi et al. [41]	Personally owned/shared-use AV	Focus groups, Online survey	CFA, SEM	TR, Co, PSa, PU, PEU → INT	0.91 0.77
Dirsehan & Can [42]	AV	Online survey	SEM	TR, sustainability concerns, PU, PEU → INT	0.57
Zhang et al. [56]	AV	Interview	SEM	PEU, PU, PSR, PPR → TR → ATT → INT	0.56, 0.67, 0.61
Wu et al. [58]	AV	Online survey	SEM	Environmental concern, Green perceived usefulness, PEU → INT	n/a
Lee et al. [57]	AV	Online survey	PLS-SEM	SE, RA, Psychological ownership, PR, PU, PEU → INT	0.52
Herrenkind et al. [30]	ASB	Online survey	PLS-SEM	EA, Openness to Shared Use, PPR, TR, PEn, RA, PrE, Residence, Family Budget, Education, Social Network → INT	0.52
Herrenkind et al. [29]	ASB	Qualitative research, interview; revealed preference	CFA, SEM	TR, LOC, PPR, EA, PI, image, SN, PEn, RA, PrE, PU, PEU, ATT → INT	n/a
Xu et al. [24]	AV	Field experiment	SEM	TR → PU, PEU, PSa → INT Willingness to re-ride	0.55 0.40
Panagiotopoulos & Dimitrakopoulos [55]	AV	Online survey	Multiple linear regression	PU, PEU, TR, SI → INT	0.44
Buckley et al. [54]	AV	Interview, revealed preference	bivariate correlations, Hierarchical regression	TR, ATT, SN, PBC → INT TR, PU, PEU → INT	0.49 0.44

Note 1. R^2^ indicates exogenous construct combined effect on the endogenous construct, and ranges from 0 to 1, where higher values indicate higher levels of prediction accuracy. Note 2. AV: Autonomous vehicle; ASB: Autonomous shuttle bus; PU: Perceived usefulness; PEU: Perceived ease of use; ATT: Attitudes; INT: Usage intention; TR: Trust; SI: Social influence; SN: Subjective norm; LOC: Locus of control/Desirability of control; RA: Relative advantage; PI: Personal/Consumer Innovativeness; Co: Compatibility; PEn: Perceived enjoyment; PrE: Price evaluation; PSa: Perceived safety; PSR: perceived safety risk; PPR: Perceived privacy risk/concerns; SE: Self-efficacy; PR: perceived risk; DIT: Diffusion of innovation.

**Table 2:** Research framework constructs with measurement indicators.

Construct	Measure	Source
Perceived Service Quality (PSQ)	Punctuality (on-time performance) ** Privacy (sharing the shuttle space with other passengers) ** Comfort (ease of entrance and exit from the vehicle/stations) ** Affordability (fare price) ** Safety on board (regarding accidents) ** Flexibility (frequency or number of daily services) ** Convenience (Individual space available inside the vehicle) ** Speed (getting places quicker) **	[61]
Perceived Relative Advantages (PRA)	I believe that ASBs will be safer than conventional shuttles. * I believe that ASBs will be more efficient than conventional shuttles. * ASBs can reduce the need for conventional shuttles. * ASBs can reduce traffic congestion and pollutant emissions compared with conventional shuttles. * There will be fewer driver errors, in the case of using ASBs. * ABSs can allow better access to my intended destinations than other available travel modes. *	Self-developed, where items were from [29,30]
Perceived Risks (PR)	Unreliable technology (trip interruption) * Traffic safety on board (regarding accidents) * AVs won’t respond in dangerous situations *	Modified from [83]
Perceived Ease of Use (PEU)	I believe it would be easy for me to understand/learn how to book a ride. * I believe it would be easy to learn how to interact with ASBs. * I believe it would be easy to learn how to travel in an ASB. *	Modified from [27]
Perceived Usefulness (PU)	Riding in ASBs can reduce the stress of driving. * Using ASBs can increase my living and working productivity by reducing the time I spend driving. * I can see more possibilities for my mobility with ASBs. * ASB transport services can serve my travel needs well. * ASB transport service can be a good mobility solution for people who are unable to drive like disabled persons or the elderly. *	Self-developed, where items were from [83]
Attitude Toward Use (ATT)	I believe that ASBs will be more attractive to use than conventional shuttles. * I have a positive attitude toward ASBs. *	Modified from [88]
Intention to Use (INT)	If shuttles become available, I will give priority to using them over using a car. * I would be happy to ride in an ASB. *	Modified from [89]

Notes: * Response Anchors: strongly disagree (1)/strongly agree (5). ** Response Anchors: very unlikely (1)/very likely (5).

**Table 3:** Demographic attributes and collinearity tolerances of predictor variables.

Predictor Variable	Category	Frequency (*n* = 300)	Distribution (%)	Collinearity (VIF)
Gender	Male	105	35.0	1.271
	Female: 195	195	65.0	
Age	18–35	108	36.0	1.965
	36–50	53	17.7	
	51–65	58	19.3	
	66 or higher	81	27.0	
Education	High School	109	36.3	1.104
	Vocational	109	36.3	
	Tertiary	82	27.4	
Employment	Retired, Homemaker, or Not Employed	138	46.0	1.531
	Part-time or Casual Employed	72	24.0	
	Full-time or Self Employed	90	30.0	
Household income	Nil to $15,599	37	12.3	1.202
	$15,600 to $31,199	43	14.3	
	$31,200 to $51,999	55	18.4	
	$52,000 to $77,999	65	21.7	
	$78,000 to $103,999	54	18.0	
	$104,000 or more	46	15.3	
Residential location	Peri-urban	200	67.0	1.151
	Urban	100	33.0	
Household size	1	66	22.0	1.355
	2	119	39.7	
	3	42	14.0	
	4	43	14.3	
	5 or more	30	10.0	

**Table 4:** Measurement model evaluation process [103].

Criterion	Description
$\mathrm{Composite}\;\mathrm{reliability}\;(\rho_{c})$	The composite reliability is a measure of internal consistency and must not be lower than 0.6. $\rho_{c}={\left(\sum \lambda_{i}\right)}^{2}/[{\left(\sum \lambda_{i}\right)}^{2}+\sum \mathrm{Var}\left(\varepsilon_{i}\right)]$, $\lambda_{i}:$ the outer (component) loading to an indicator, and $\mathrm{Var}\left(\varepsilon_{i}\right)=1-{\;\lambda }_{i}{}^{2}$ in the case of standardized indicators.
Indicator reliability	Absolute standardized outer (component) loadings should be higher than 0.7.
Average variance extracted (AVE)	$\mathrm{AVE}={\left(\sum \lambda_{i}\right)}^{2}/[{\left(\sum \lambda_{i}\right)}^{2}+\sum \mathrm{Var}\left(\varepsilon_{i}\right)]$, where $\lambda_{i}$ is the component loading to an indicator and $\mathrm{Var}\left(\varepsilon_{i}\right)=1-{\;\lambda }_{i}{}^{2}\;$ in the case of standardized indicators. The average variance extracted should be higher than 0.5.
Fornell–Larcker criterion	To ensure discriminant validity, the AVE of each latent variable should be higher than the squared correlations with all other latent variables. Thereby, each latent variable shares more variance with its block of indicators than with another latent variable representing a different block of indicators.
Cross-loadings	Cross-loadings offer another check for discriminant validity. If an indicator has a higher correlation with another latent variable than with its respective latent variable, the appropriateness of the model should be reconsidered.

**Table 5:** Construct reliability and validity results (CA, CR, AVE, and inter-construct correlations).

	Coefficient Alpha (CA > 0.7)	Composite Reliability (CR > 0.7)	Average Variance Extracted (AVE > 0.5)	ATT	INT	PEU	PRA	PR	PSQ	PU
ATT	0.716	0.876	0.779	**0.882**						
INT	0.712	0.874	0.776	0.575	**0.881**					
PEU	0.810	0.887	0.724	0.598	0.504	**0.851**				
PRA	0.891	0.916	0.646	0.490	0.354	0.587	**0.804**			
PR	0.873	0.922	0.797	−0.300	−0.291	−0.149	−0.113	**0.893**		
PSQ	0.886	0.908	0.555	0.522	0.442	0.475	0.352	−0.276	**0.745**	
PU	0.897	0.924	0.709	0.586	0.524	0.688	0.506	−0.132	0.469	**0.842**

Bolded numbers: square root of AVE.

**Table 6:** Factor loadings (in bold) and cross-loadings.

Latent Construct	Loadings > 0.6								VIF
	Indicator	ATT	INT	PEU	PR	PRA	PSQ	PU	
Attitude	ATT1	**0.872**	0.474	0.518	0.479	−0.282	0.409	0.475	1.452
	ATT2	**0.893**	0.538	0.538	0.391	−0.250	0.508	0.556	1.452
Usage Intention	INT1	0.565	**0.895**	0.480	0.388	−0.219	0.435	0.462	1.441
	INT2	0.442	**0.867**	0.405	0.227	−0.300	0.340	0.463	1.441
	PEU1	0.492	0.364	**0.788**	0.461	−0.195	0.365	0.465	1.547
Perceived Ease of Use	PEU2	0.411	0.401	**0.879**	0.442	−0.163	0.393	0.620	2.132
	PEU3	0.608	0.506	**0.883**	0.581	−0.046	0.447	0.653	1.940
Perceived Risks	PR1	−0.292	−0.254	−0.156	−0.124	**0.903**	−0.258	−0.127	2.455
	PR2	−0.276	−0.248	−0.144	−0.099	**0.899**	−0.232	−0.116	2.461
	PR3	−0.235	−0.279	−0.097	−0.078	**0.877**	−0.249	−0.110	2.149
Perceived Relative Advantages	PRA1	0.315	0.219	0.437	**0.822**	−0.066	0.238	0.389	2.709
	PRA2	0.335	0.223	0.411	**0.788**	−0.111	0.267	0.387	2.366
	PRA3	0.392	0.283	0.522	**0.819**	−0.067	0.314	0.415	2.251
	PRA4	0.420	0.316	0.432	**0.772**	−0.105	0.307	0.372	1.923
	PRA5	0.402	0.299	0.475	**0.855**	−0.071	0.298	0.414	2.675
	PRA6	0.468	0.340	0.529	**0.764**	−0.118	0.264	0.446	1.701
Perceived Service Quality	PSQ1	0.212	0.242	0.207	0.109	−0.124	**0.637**	0.209	1.774
	PSQ2	0.503	0.454	0.407	0.308	−0.271	**0.798**	0.477	2.018
	PSQ3	0.392	0.306	0.376	0.279	−0.263	**0.758**	0.330	2.043
	PSQ4	0.476	0.374	0.425	0.347	−0.262	**0.825**	0.417	2.329
	PSQ5	0.277	0.264	0.243	0.115	−0.145	**0.708**	0.270	1.965
	PSQ6	0.414	0.294	0.356	0.284	−0.168	**0.765**	0.351	2.146
	PSQ7	0.311	0.274	0.354	0.241	−0.152	**0.692**	0.302	1.871
	PSQ8	0.400	0.350	0.383	0.311	−0.195	**0.760**	0.339	1.983
Perceived Usefulness	PU1	0.483	0.505	0.605	0.411	−0.089	0.390	**0.860**	2.828
	PU2	0.481	0.398	0.570	0.438	−0.051	0.315	**0.851**	2.760
	PU3	0.508	0.474	0.596	0.469	−0.120	0.417	**0.854**	2.390
	PU4	0.514	0.436	0.536	0.389	−0.163	0.456	**0.825**	2.120
	PU5	0.481	0.385	0.587	0.420	−0.131	0.394	**0.819**	2.098

**Table 7:** Heterotrait–Monotrait (HTMT) values.

	ATT	INT	PEU	PRA	PR	PSQ	PU
ATT							
INT	0.798						
PEU	0.777	0.652					
PRA	0.608	0.431	0.678				
PR	0.380	0.373	0.187	0.126			
PSQ	0.625	0.537	0.541	0.374	0.301		
PU	0.729	0.654	0.798	0.562	0.149	0.506	

**Table 8:** Structural model assessment process (derived from [103]).

Criterion	Description
$R^{2}$ of endogenous latent variables	$R^{2}$ values of 0.67, 0.33, or 0.19 for endogenous latent variables in the inner path model are described as substantial, moderate, or weak [116].
Estimates for path coefficients	The estimated values for path relationships in the structural model should be evaluated in terms of the sign, magnitude, and significance (the latter via bootstrapping).
$\mathrm{Prediction}\;\mathrm{relevance}\;(Q^{2}$)	$\mathrm{The}\;Q^{2}$ is calculated based on the blindfolding procedure: $Q^{2}=1-(\underset{D}{\sum }{\mathrm{SSE}}_{D})/(\underset{D}{\sum }{\mathrm{SSO}}_{D}),$ D: the omission distance, SSE: the sum of squares of prediction errors, and SSO: the sum of squares of observations. $Q^{2}>0:$ give evidence that the observed values are well reconstructed and that the model has predictive relevance, ($Q^{2}<0$ indicates a lack of predictive relevance).

**Table 9:** Structural model evaluation results.

Proposed Hypotheses		Effect	β	T-Value	*p*-Value	Results
H1	Perceived Ease of Use (PEU) + → Attitude (ATT)	+	0.23	2.92	0	Supported
H2	Perceived Usefulness (PU) + → Attitude (ATT)	+	0.24	3.30	0	Supported
H3	Perceived Ease of Use (PEU) + → Perceived Usefulness (PU)	+	0.53	7.94	0	Supported
H4	Perceived Usefulness (PU) + → Usage Intention (INT)	+	0.26	3.76	0	Supported
H5	Attitude (ATT) + → Usage Intention (INT)	+	0.32	4.43	0	Supported
H6	Perceived Service Quality (PSQ) + → Perceived Usefulness (PU)	+	0.17	3.09	0	Supported
H7	Perceived Service Quality (PSQ) + → Attitude (ATT)	+	0.21	3.34	0	Supported
H8	Perceived Service Quality (PSQ) + → Usage Intention (INT)	+	0.11	1.82	0.07	Not supported
H9	Perceived Relative Advantage (PRA) + → Perceived Usefulness (PU)	+	0.14	2.45	0.01	Supported
H10	Perceived Relative Advantage (PRA) + → Attitude (ATT)	+	0.15	2.64	0.01	Supported
H11	Perceived Relative Advantage (PRA) + → Usage Intention (INT)	+	0.01	0.19	0.85	Not supported
H12	Perceived Risks (PR) + → Perceived Usefulness (PU)	+	0.01	0.27	0.78	Not supported
H13	Perceived Risks (PR) - → Attitude (ATT)	−	−0.16	3.65	0	Supported
H14	Perceived Risks (PR) - → Usage Intention (INT)	−	−0.13	2.75	0.01	Supported

Note. β: Path coefficient, t-value: Significance, *p*-value: Significance.
